# Supplementary material for: Management of Donkeys in Assisted Interventions: A Snapshot
Source: Animals (Basel). 2024 Feb 21;14(5):670. doi: 10.3390/ani14050670 (PMC10930421; doi:10.3390/ani14050670)
Supplement: Supplementary file 1 [file animals-14-00670-s001.zip › animals-2876923-supplementary.pdf]

Table S1. Questionnaire on Donkey management in Assisted-Intervention Facilities

**1. General**	Yes	No
Specialized center in Animal Assisted Education and Therapy according to the national guidelines for Animal-Assisted Interventions (AAI)		
All kinds of AAI (activities with recreational, educational and therapeutic purposes)		
Activities with recreational purpose (AAA) only		
Personnel with qualifications relevant to donkey's welfare<br>Ex. Master degree in veterinary medicine<br>Degree in animal husbandry<br>Animal Welfare Specializations		
Staff responsible for cleaning, feeding and animal supervision		
Regular collaboration with a veterinarian trained in AAI<br>(once a month or more often)		
Sporadic collaboration with a veterinarian trained in AAI<br>(less than once a month)		
Absence of collaboration with a veterinarian trained in AAI		
**2. Housing**		
Paddock with shelter (tree walls)		
Stall with paddock and regulated access		
Animal supplies		
Home-made solutions		
Ground stability or tendency of the paddock's terrain to remain dry, not to become muddy during bad weather		
Grass-covered paddok<br>(to be assessed at the time of the visit)		
Presence of shaded area different from that provided by the shelter or the stall<br>(to be assessed at the time of the visit)		
Presence of environmental enrichment<br>(to be assessed at the time of the visit)		
**3. Feeding**		
Only hay as forage		
Only straw as forage		
Hay and straw as forage (mostly hay)		
Straw and hay as forage (mostly straw)		
Free access to pasture all year round		
Access to pasture regulated on a seasonal basis		
Weekly supplementation of donkeys' diet with edible plants' shrubs		
Weekly supplementation of donkeys' diet with shrubs fruits and vegetables		
Fruits and vegetables administered as reward during interaction with users		
Mineral supplementation<br>Ex. salt in blocks		
Rationed forage		
Forage always available (administration *ad libidum*)		
Forage bale feeder		
Feeding troughs		

Suspended net		
Forage on the ground		
**3. Health**		
Weight monitoring: weight measurement or estimation through chest circumference and height at the withers (once a month or more often)		
Dental check-up and corrective interventions (once a year)		
Hoof check and potential trimming (once every three month or more often)		
Hoof check and potential trimming (less than once every three month)		
Vaccination for Equine Influenza and Tetanus (once a year)		
Vaccination for Nile Disease (once a year)		
No vaccinations		
Endoparasites monitoring through fecal exam and selective treatment (once a year)		
Blind administration of antiparasitic drug (once or twice a year)		
